# Pentraxin 3 promotes airway inflammation in experimental asthma

**DOI:** 10.1186/s12931-020-01499-6

**Published:** 2020-09-16

**Authors:** Pengfei Gao, Kun Tang, Yanjiao Lu, Zhenli Huang, Shanshan Wang, Meijia Wang, Jianmiao Wang, Jianping Zhao, Jungang Xie

**Affiliations:** 1 Department of Respiratory and Critical Care Medicine, Key Laboratory of Pulmonary Diseases of Health Ministry, National Clinical Research Center of Respiratory Disease, Tongji Hospital of Tongji Medical College, Huazhong University of Science and Technology, Wuhan, Hubei China; 2 Department of Respiratory and Critical Care Medicine, The First Affiliated Hospital, College of Clinical Medicine, Henan University of Science and Technology, Luoyang, Henan China

**Keywords:** Asthma, Airway inflammation, Eosinophil, Neutrophil, PTX3

## Abstract

**Background:**

Pentraxin 3 (PTX3) regulates multiple aspects of innate immunity and tissue inflammation. Recently, it has been reported that PTX3 deficiency enhances interleukin (IL)-17A–dominant pulmonary inflammation in an ovalbumin (OVA)-induced mouse asthma model. However, whether PTX3 treatment would provide protection against allergic airway inflammation has not been clearly elucidated. The goal of this study was to further investigate the effect of recombinant PTX3 administration on the phenotype of asthma.

**Methods:**

C57BL/6 J mice were sensitized and challenged with OVA to induce eosinophilic asthma model, as well as sensitized with OVA plus LPS and challenged with OVA to induce neutrophilic asthma model. We evaluated effect of recombinant PTX3 on asthma phenotype through both asthma models. The bronchoalveolar lavage fluid (BALF) inflammatory cells and cytokines, airway hyperresponsiveness, and pathological alterations of the lung tissues were assessed.

**Results:**

In both eosinophilic and neutrophilic asthma models, PTX3 treatment provoked airway hyperresponsiveness, concomitant with increased inflammatory cytokines IL-4, IL-17, eotaxin, and transforming growth factor (TGF)-β1 and aggravated airway accumulation of inflammatory cells, especially eosinophils and neutrophils. In histological analysis of the lung tissue, administration of PTX3 promoted inflammatory cells infiltration, mucus production, and collagen deposition. In addition, PTX3 also significantly enhanced STAT3 phosphorylation in lung tissue.

**Conclusion:**

Our results show that exogenous PTX3 can exacerbate multiple asthmatic features by promoting both eosinophils and neutrophils lung infiltration and provide new evidence to better understand the complex role of PTX3 in allergic airway inflammation.

## Introduction

Asthma is a heterogeneous disease characterized by chronic airway inflammation, and many cells of the innate and adaptive immune systems act together with epithelial cells to cause airway hyperresponsiveness, mucus overproduction, and airway wall remodeling [1]. Classically, inflammation including T helper-2 (Th2) cells and Th2-associated cytokines, as well as eosinophils play central roles in asthma pathogenesis [2]. However, there is mounting evidence that some asthmatic patients do not exhibit typical asthmatic features compared with classic allergic asthma [3]. In fact, approximately half of asthmatic patients present with non-eosinophilic airway inflammation [4].

The long pentraxin 3 (PTX3) is a prototypic pattern recognition receptor, highly conserved in evolution, which has served as a representative molecular to dissect recurrent themes in humoral innate immunity [5]. PTX3, produced locally in response to inflammatory stimuli, is the long member of the pentraxin family together with C-reactive protein and the serum amyloid P component [5]. Growing amount of studies, including mouse and human genetics, suggest that PTX3 plays essential non-redundant roles in innate immunity and inflammation, as well as in tissue remodeling [6]. Previously, PTX3 was shown to be protective in models of tissue injury mediated by infection, LPS treatment, and sterile inflammation [7–11]. However, PTX3 could also potentially lead to harmful consequences under certain circumstances, such as ischemia and reperfusion injury [12], *Ross River virus* infection [13], and vascular endothelial dysfunction [14]. Alternatively, PTX3 can exert potential contradictory roles in inflammation regulation, antimicrobial resistance, and disease pathogenesis, depending on the tissue context, cellular source, and levels of production.

Elevated PTX3 expression in asthmatic airways was firstly reported by Zhang and colleagues [15]. Sputum PTX3 levels were also shown to be increased in children with asthma and reported to be a candidate biomarker for evaluation of airway inflammation and remodeling [16]. Licari and colleagues also reported elevated plasma PTX3 concentrations in children with asthma, but no significant correlation with clinical parameters was observed [17]. This is partly due to that PTX3 concentration, particularly at systemic level, is influenced by its various cellular source. Recently, PTX3 deficiency was shown to result in augmented airway hyperresponsiveness (AHR), mucus production, and IL-17-dominant pulmonary inflammation in an OVA-induced mouse asthma model [18]. In view of the complexity role of PTX3 in disease pathogenesis as described above, it was necessary to identify whether PTX3 treatment would provide protection against unbalanced inflammatory responses in asthma, and elucidate the exact role of PTX3 in the disease pathogenesis.

Our group previously reported differential expression of pentraxins including PTX3 in asthmatic patients, which partly reflected heterogeneity of innate immunity in asthma [19]. Accordingly, both eosinophilic and neutrophilic asthma models were applied to comprehensively evaluate the effect of exogenous PTX3 on the phenotype of asthma, by assessing AHR, inflammatory cell infiltration, cytokine production, mucus production and collagen disposition.

## Methods

### Animals and experimental protocol

**Fig. 1:**
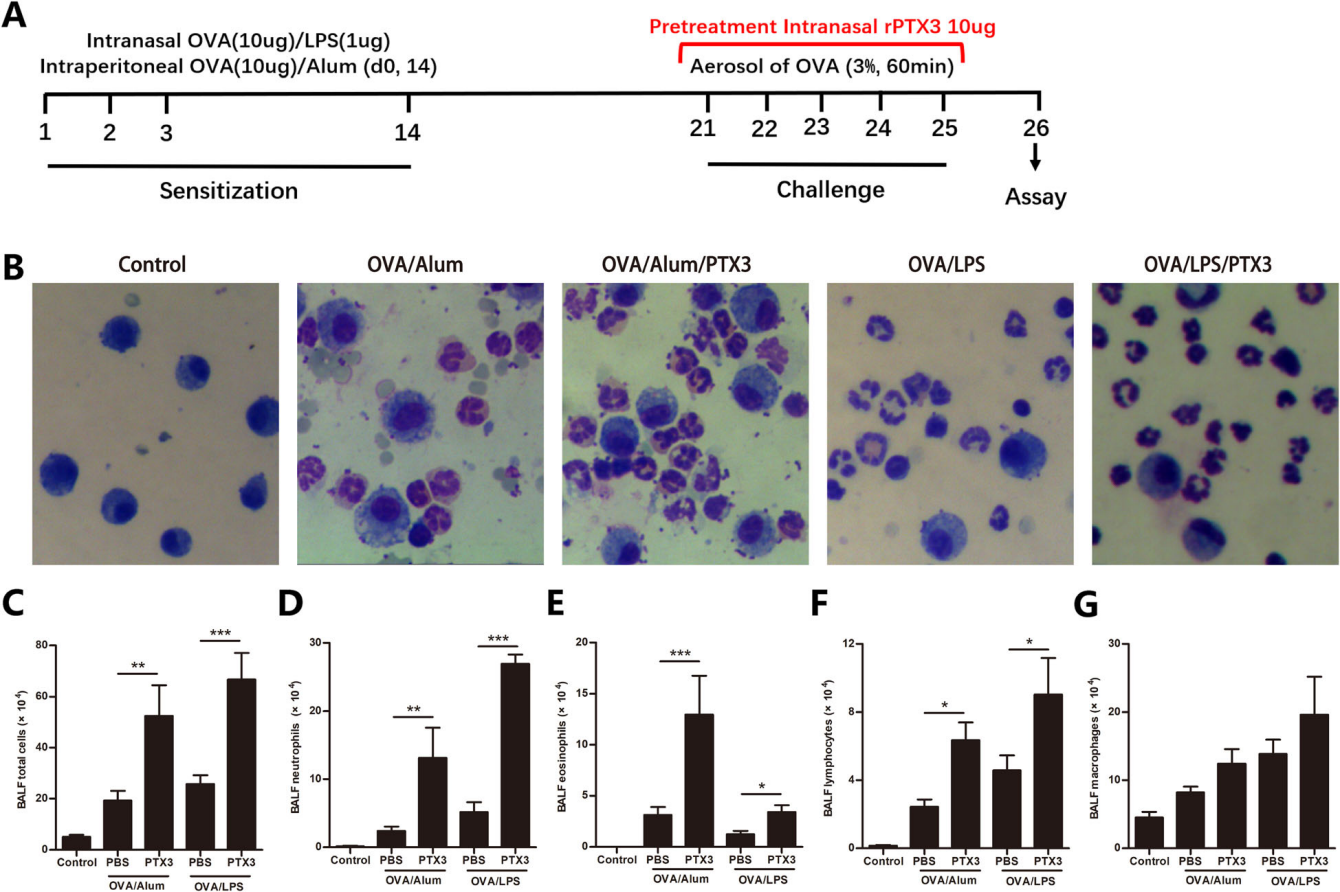
Effects of rPTX3 treatment on bronchoalveolar lavage fluid (BALF). **a** Timeline of the protocol for establishing neutrophilic and eosinophilic asthma model. In general, mice were sensitized in the presence of either OVA/LPS or OVA/Alum, followed by exposure to OVA aerosols for 5 consecutive days. One hour before challenge, mice were administrated intranasally with rPTX3. One day later, BALF was isolated to determine differential cell counts and cytokines levels. **b** Representative cytospin images of Liu’s solution staining. **c** Total cells, **d** neutrophils, **e** eosinophils, **f** lymphocytes, and **g** macrophages were counted in BALF. Data are shown as the mean ± SEM (*n* = 6–10), *P < 0.05, **P < 0.01, and ***P < 0.001. Data were analysed using one-way analysis of variance (ANOVA) followed by the Tukey-Kramer post hoc test for multiple comparisons

For the neutrophilic OVA/LPS model, 8–10 weeks old female C57BL/6 mice (HFK bioscience, Beijing, China) were sensitized intranasally with 10 μg OVA grade V (Sigma-Aldrich, St Louis, Mo, USA), and 1 μg LPS (Sigma-Aldrich) in saline on days 1, 2, 3, and 14. For the eosinophilic OVA/Alum model, mice were intraperitoneally sensitized on days 1 and 14 with 10 μg OVA grade V emulsified in 1 mg aluminum hydroxide (Pierce Chemical, Rockford, Ill) in a total volume of 200 μL. From days 21 to 25, all mice were challenged daily for 60 min with an aerosol of 3% OVA grade II (Sigma-Aldrich) in saline (or with saline as a control) (Fig. 1a), as previously described with some modifications [20]. All mice were killed for analysis 24 h after the challenge. Mice were anesthetized with intraperitoneal injections of 200 μL 1% pentobarbital sodium solution. We measured pulmonary resistance in response to a range of concentrations of inhaled methacholine using the forced oscillation technique with the FlexiVent system (SCIREQ, Montreal, Quebec, Canada). The lungs were washed 2 times with 0.7 mL of PBS, and bronchoalveolar lavage fluid (BALF) collected from each mouse was centrifuged at 1000 rpm for 10 min, and the supernatants were stored at − 80 °C until use. Total cell numbers were counted with a hemocytometer. Smears of BALF cells were prepared by using a cytospin and stained with Liu’s solution (Promoter biotech, Wuhan, China) to examine cell differentials in a blinded manner. The left lungs were fixed in 4% formaldehyde for histological analysis, and the right lungs were stored at − 80 °C until use. Animal experiments were approved by the ethics committee of Tongji Hospital, Huazhong University of Science and Technology.

### Administration of recombinant mouse PTX3

Recombinant mouse PTX3 were purified under endotoxin-free conditions from the supernatants of stably transfected CHO cells constitutively expressing the proteins in Qianjinuo Biotech, Nanjing, China, as described [21]. Recombinant PTX3 contained < 0.125 endotoxin units/ml, as checked by the Limulus amebocyte lysate assay (BioWhittaker, Walkersville, MD). The rPTX3 was dissolved in sterile PBS and diluted to 200 μg/mL, and then 50 μL of solution, corresponding with an dose of 10μg, was delivered to mice intranasally 1 h before challenge for 5 consecutive days. An equal volume of PBS was used as a vehicle control.

### Enzyme-linked immunosorbent assay

IL-4, IL-13, IL-17, IFNγ, eotaxin, TGFβ1 (R&D Systems, Minneapolis, MN) levels in BALF and serum IgE (Biolegend, SanDiego, CA) were quantified by enzyme-linked immunosorbent assay (ELISA) according to the manufacturers’ instructions. Sensitivities for the IL-4, IL-13, IL-17, IFNγ, eotaxin, TGFβ1 and IgE assays were 2, 1.5, 5, 2, 3, 15.4 pg/mL, and 0.1 ng/mL, respectively.

### Western blotting

Total proteins of mouse lung tissue were extracted in lysis buffer containing phosphatase inhibitors using a BCA protein-assay kit (Bioyear Biotechnology, Wuhan, China) to measure concentrations. Once separated by 10% SDS-PAGE, the proteins were transferred to polyvinylidene difluoride membranes (Merck Millipore) and blocked (5% evaporated milk in Tris-buffered saline containing 0.05% Tween 20 [TBST]) for 1–2 h, followed by overnight incubation with primary antibodies at 4 °C. Corresponding primary antibodies were applied: anti-pSTAT3 (1:1000; Cell Signaling Technology, Danvers, MA, USA), anti-STAT3 (1:1000, Cell Signaling Technology), anti-Collagen I (1:1000, Servicebio, Wuhan, China) and anti-β-actin antibody (1:4000, ABclonal Biotechnology). Membranes were then washed, and incubated for 1 h at room temperature with secondary antibodies conjugated to HRP (1:4000; Bioyear Biotechnology). Protein bands were imaged (ChemiDoc XRS + system; Bio-Rad, Hercules, CA, USA) and quantitatively analyzed (Image Lab; Bio-Rad).

### Immunofluorescence

Paraffin-embedded lung sections were stained with primary MPO (1:50, Abcam), EPO (1:100, Bioss), and CD3 (1:100, Abcam), and nuclei were counter-stained with DNA with 4′,6 diamidino-2-phenylindole (DAPI). In order to quantify the recruitment of inflammatory cells, sections were visualized at magnification × 200 and neutrophils (MPO-positive), eosinophils (EPO-positive) and lymphocytes (CD3-positive) staining was assessed by mean fluorescence intensity (MFI) analysis in 5 different fields for each section using ImageJ software.

### Histological evaluation

Paraffin-embedded 5-μm lung sections were stained with hematoxylin and eosin. The severity of peribronchial inflammation was scored by a blinded observer using the following features: 0, normal; 1, few cells; 2, a ring of inflammatory cells 1 cell layer deep; 3, a ring of inflammatory cells 2–4 cells deep; 4, a ring of inflammatory cells of > 4 cells deep [22]. Lung sections were also stained with periodic acid Schiff (PAS) staining (Goodbio technology, Beijing, China) for detection of mucus-containing cells. The numerical scores for the abundance of PAS-positive cells in each airway were determined as follows: 0, < 0.5% PAS-positive cells; 1, 5–25%; 2, 25–50%; 3, 50–75%; 4, > 75% The percentage of PAS-positive mucus containing cell in each airway were determined [22]. To quantitate remodeling changes, we measured total lung collagen I, an index of collagen deposition.

### Statistical analysis

Data are expressed as means ± SEMs. Statistical analysis was performed using one-way analysis of variance (ANOVA) followed by the Tukey-Kramer post hoc test for multiple comparisons. We used GraphPad Prism 5 (GraphPad, San Diego, CA, USA), and *P* < 0.05 was considered statistically significant.

## Results

### Recombinant PTX3 enhanced cells influx in lung

**Fig. 2:**
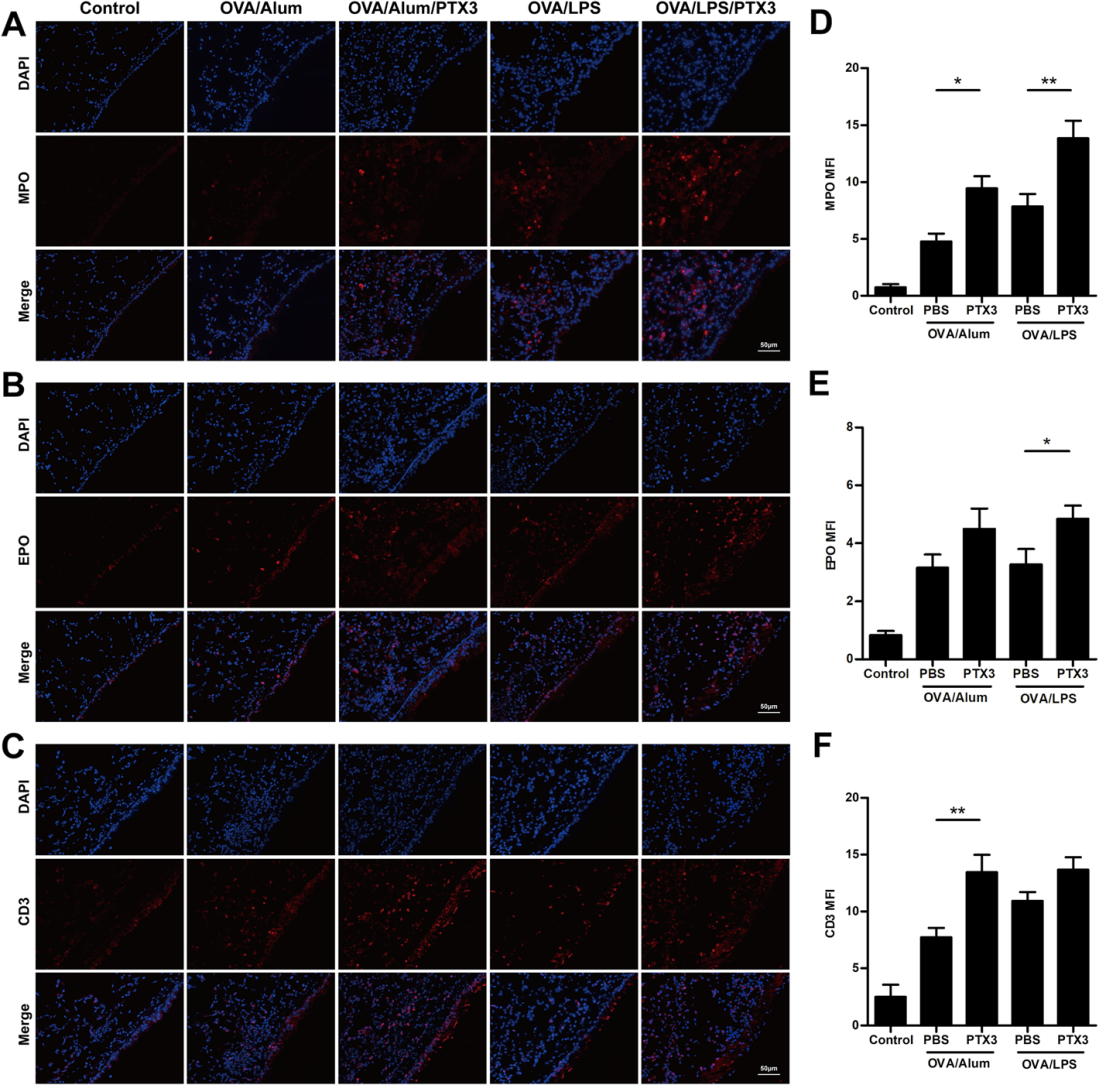
Effects of rPTX3 treatment on inflammatory cell infiltration into lung tissue. Representative pictures of MPO staining (**a**), EPO staining (**b**), and CD3 staining (**c**). The bar charts demonstrate the differences between neutrophils (MPO-positive) MFI (**d**), eosinophils MFI (EPO-positive) (**e**) and lymphocytes (CD3-positive) MFI (**f**). Data are shown as the mean ± SEM (*n* = 6–10), *P < 0.05 and **P < 0.01. Data were analysed using one-way analysis of variance (ANOVA) followed by the Tukey-Kramer post hoc test for multiple comparisons

To further understand the role of PTX3 in asthma, eosinophilic and neutrophilic asthma models were established. Immunization of mice with OVA/LPS has been reported to cause neutrophilic airway inflammation, in contrast to the eosinophilic airway inflammation provoked by OVA/Alum [20]. We confirmed that immunization with OVA, followed 3 weeks later by nebulized OVA for 5 consecutive days, leads to significant cell infiltration in the bronchoalveolar lavage fluid (BALF) compared with control mice (Fig. 1a). Asthmatic mice were intranasally administered rPTX3 or equal volume PBS before each challenge starting on day 21 (Fig. 1a). In mice sensitized to OVA/Alum, rPTX3 treatment significantly enhanced the immune cell influx (Fig. 1b-c), particularly neutrophils (Fig. 1d), eosinophils (Fig. 1e), and lymphocytes (Fig. 1f). BALF cellular infiltration in response to OVA/LPS sensitization were also enhanced by rPTX3 administration (Fig. 1b-f). Correspondingly, exacerbated inflammatory cell infiltration into lung tissue was also observed in both asthma models (Fig. 2).

### Recombinant PTX3 enhanced AHR

**Fig. 3:**
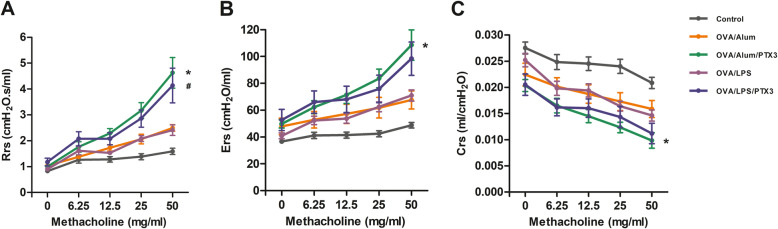
Effects of rPTX3 treatment on airway hyperresponsiveness (AHR). Respiratory system resistance (**a**), elastance (**b**), and compliance (**c**) were measured 24 h after final challenge by FlexiVent. Data are shown as the mean ± SEM (*n* = 6–10), *p ≤ 0.05 compared to OVA/Alum and ^#^*p* ≤ 0.05 compared to OVA/LPS. Data were analysed using one-way analysis of variance (ANOVA) followed by the Tukey-Kramer post hoc test for multiple comparisons

OVA sensitization and subsequent challenge is known to lead to the development of AHR. Therefore, we also assessed the effect of treatment with rPTX3 on airway responses in the OVA/Alum and OVA/LPS models. We measured resistance, compliance and airway tissue elasticity to aerosolized methacholine (MCh) by an invasive method 24 h after the last challenge with the flexiVent setup. After Mch inhalation to provoke bronchial hyperresponsiveness, we demonstrated rPTX3 treatment caused significant enhancement in resistance (Fig. 3a) and elasticity (Fig. 3b) parameters, and corresponding reduction in airway tissue compliance (Fig. 3c) in mice sensitized by OVA/Alum. Similar results were observed in OVA/LPS model (Fig. 3a-c), though not statistically significant in elastance and compliance.

### Effect of Recombinant PTX3 treatment on cytokine profiles in BALF

**Fig. 4:**
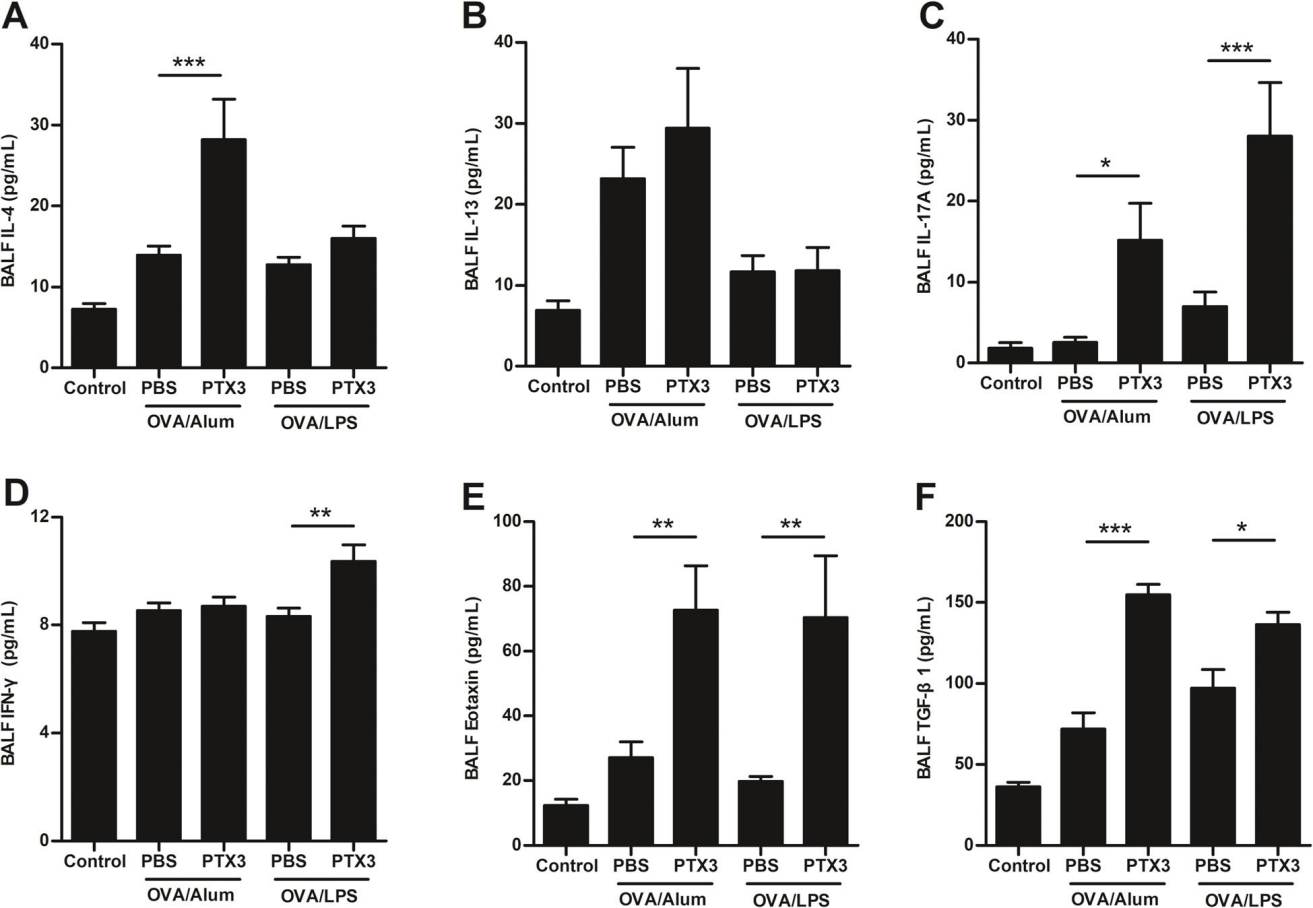
Effects of rPTX3 treatment on cytokine production in BALF. IL-4 (**a**), IL-13 (**b**), IL-17A (**c**), IFNγ (**d**), Eotaxin (**e**) and TGFβ (**f**) were measured in BALF. Data are shown as the mean ± SEM (*n* = 6–10), *P < 0.05, **P < 0.01, and ***P < 0.001. Data were analysed using one-way analysis of variance (ANOVA) followed by the Tukey-Kramer post hoc test for multiple comparisons

Cytokine levels in BALF were measured by ELISA. In mice sensitized to OVA/Alum, treatment with rPTX3 before challenge significantly increased BALF IL-4 levels (Fig. 4a), and IL-13 (Fig. 4b) secretion in BALF also tended to be enhanced by PTX3. Notably, IL-17A (Fig. 4c) and eotaxin (Fig. 4e) levels in BALF, which serve as chemoattractants for neutrophils and eosinophils respectively, were substantially upregulated by rPTX3 administration in either OVA/Alum or OVA/LPS models. Additionally, levels of IFN-γ were comparable in all groups, with only slightly elevation after rPTX3 treatment in mice sensitized to OVA/LPS (Fig. 4d). Moreover, rPTX3 treatment also resulted in significantly elevated TGF-β1 (Fig. 4f) levels in BALF in both asthma models. Taken together, these results suggest that rPTX3 may upregulate asthma phenotype by augmenting the expression of inflammatory cytokines.

### Effects of Recombinant PTX3 treatment on histopathological changes

**Fig. 5:**
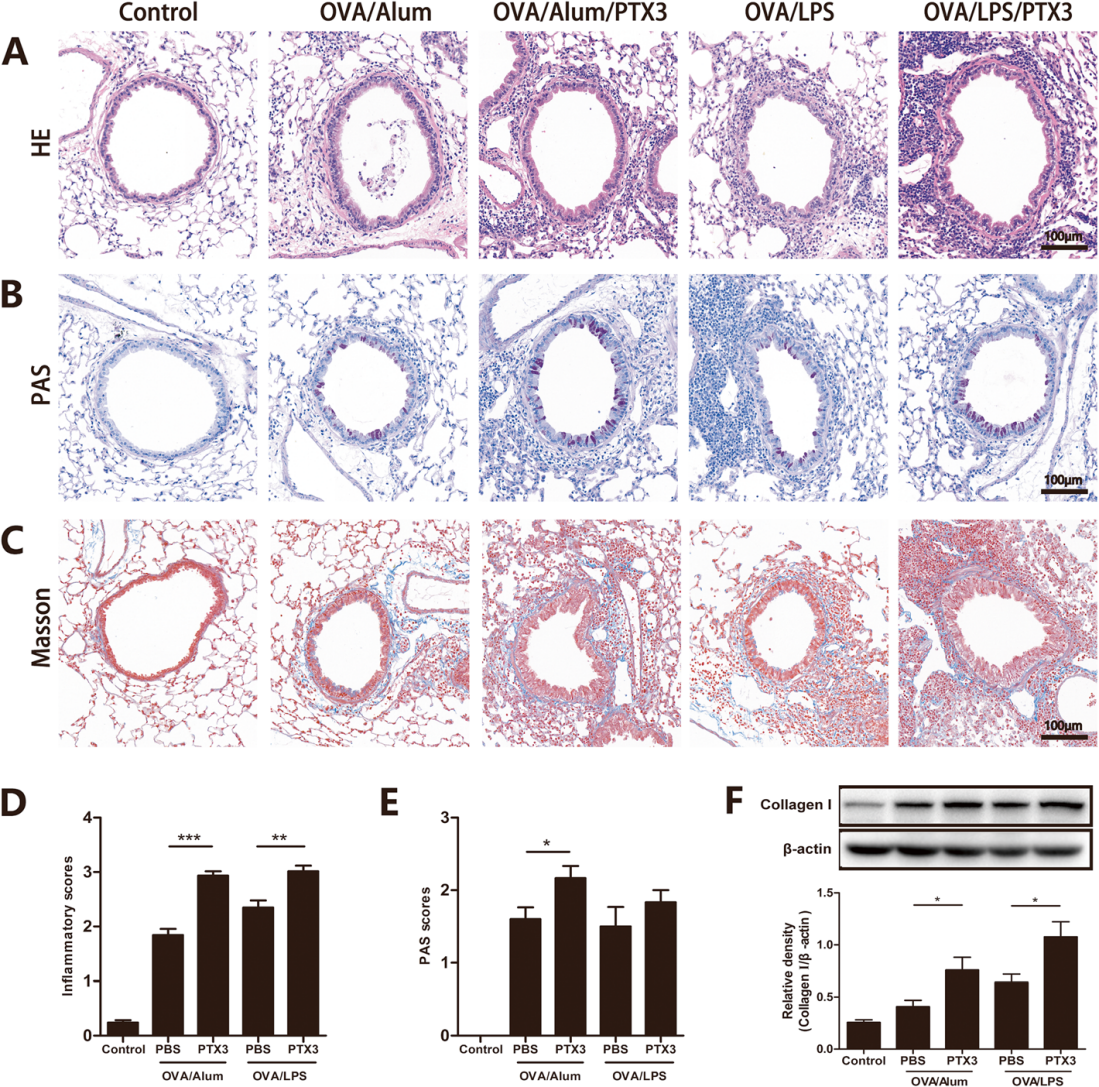
Effects of rPTX3 treatment on histopathological changes. Representative pictures of H&E staining (**a**), PAS staining (**b**), and Masson staining (**c**). The bar charts demonstrate the differences between inflammatory scores (**d**), and PAS scores (**e**). Representative western blot pictures of Collagen I in lung tissues, and protein expressions of Collagen I were quantified based on the density of the bands (**f**). Data are shown as the mean ± SEM (*n* = 6–10), *P < 0.05, **P < 0.01, and ***P < 0.001. Data were analysed using one-way analysis of variance (ANOVA) followed by the Tukey-Kramer post hoc test for multiple comparisons

Asthma is an inflammatory disease of the airways, and the inflammatory cell infiltration and inflammatory reaction in the lung tissue can directly reflect the state of an illness. In both eosinophilic and neutrophilic asthma models, besides thickening of basilar membrane, and blur of bronchiolic wall structure, the OVA-challenge groups showed elevated inflammatory cell infiltration into the lung tissue compared to the control group, and rPTX3 treatment could further exacerbate the above pathological changes (Fig. 5a), consistent with the results of inflammatory cell counting in BALF. According to PAS and masson staining, similar results were also observed in airway mucus production (Fig. 5b) and collagen deposition (Fig. 5c). Correspondingly, the semi-quantitative results indicated that rPTX3 could clearly increase the inflammation scores (Fig. 5d) and PAS scores (Fig. 5e), especially in OVA/Alum sensitized mice. To quantitate remodeling changes, we measured total lung collagen I, an index of collagen deposition. Treatment with rPTX3 obviously increased the density of collagen I in both asthma models, and the data analysis of collagen I expression also confirmed the above statement (Fig. 5f).

### Treatment with Recombinant PTX3 enhanced STAT3 phosphorylation in the lung

**Fig. 6:**
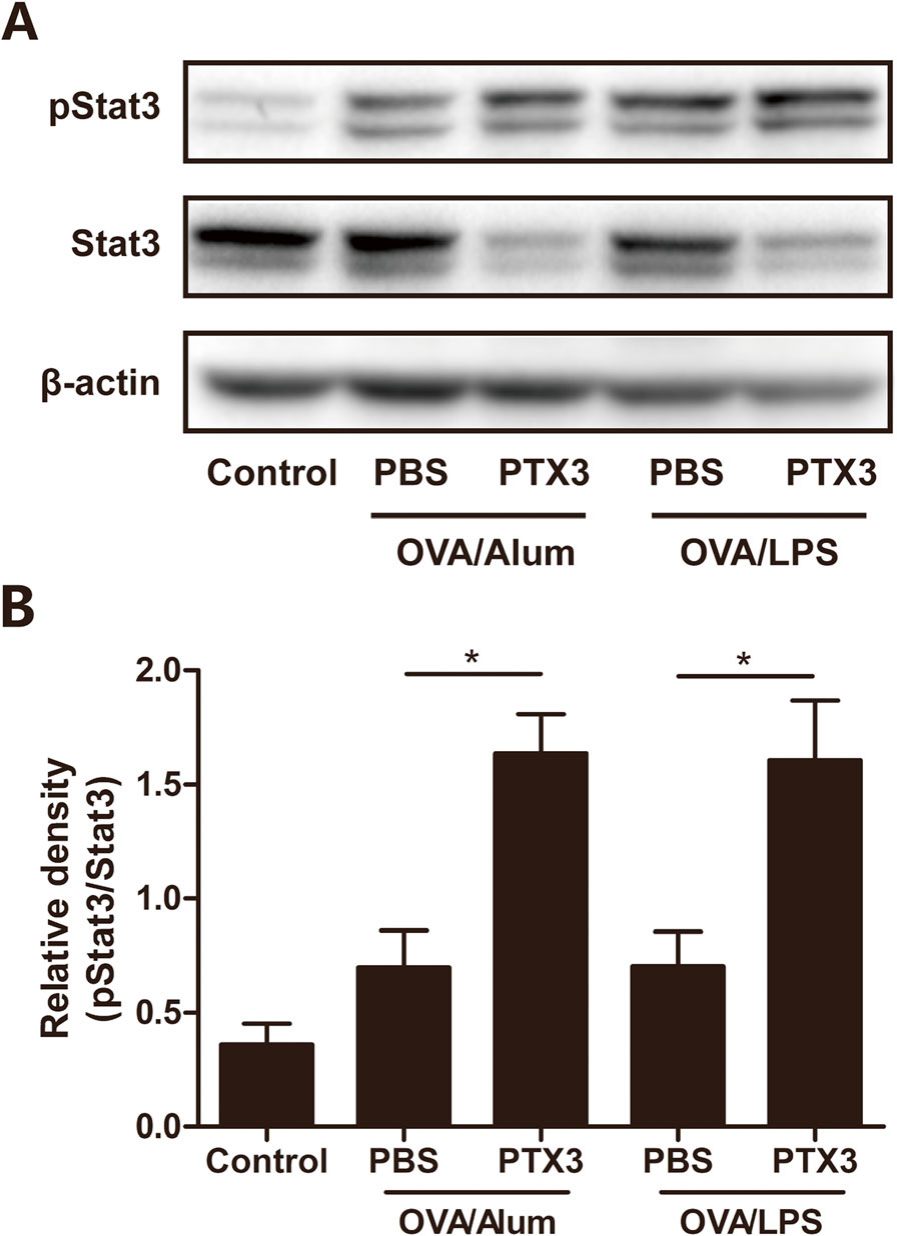
Effects of rPTX3 treatment on regulating STAT3 phosphorylation in lung tissues. Representative western blot pictures of p-STAT3 and STAT3 (**a**). Protein expressions of p-STAT3/STAT3 were quantified based on the density of the bands (**b**). Data are shown as the mean ± SEM (*n* = 4), *P < 0.05. Data were analysed using one-way analysis of variance (ANOVA) followed by the Tukey-Kramer post hoc test for multiple comparisons

Signal transducer and activator of transcription 3 (STAT3) is essential for Th17 lymphocyte development and cytokine production and its activation is linked to the development of airway inflammation [23]. The pSTAT3 bands in the lung was clearly increased by rPTX3 treatment in either OVA/Alum or OVA/LPS models (Fig. 6a), and the data analysis of phosphorylation of STAT3 also confirmed the above statement (Fig. 6b).

## Discussion

The pathophysiological roles of PTX3 have been widely investigated using genetically modified mice in various diseases, including infection, tissue injury, and cancer. It is well established that PTX3 plays an essential role in host defense, inflammation regulation, and tissue remodeling. However, the exact role of PTX3 in allergic disease context has not been fully elucidated. Balhara et al. recently reported that PTX3 deficiency resulted in upregulated asthma phenotype associated with enhanced IL-17A–dominant airway inflammation in OVA-sensitized/challenged mice [18]. Interestingly, our study showed that rPTX3 treatment exacerbated inflammatory cell infiltration, airway hyperresponsiveness, mucus hypersecretion, airway remodeling and production of inflammatory cytokine, especially IL-17A, in both eosinophilic and neutrophilic asthma models.

PTX3-induced IL-17A release may be a major contributor to elevated neutrophils in BALF in both animal models. Moreover, PTX3 has been shown to induce eotaxin-1 production in human airway smooth muscle cells in vitro [15], and here we observed increased eotaxin levels in BALF, accompanied by increase in BALF eosinophils in PTX3 treated animals in both models. In OVA/Alum model, IL-4 was also shown to increase in PTX3-treated mice, so was IL-13, though not significant, in line with the results observed in animal model of diabetic nephropathy [24]. In addition, rPTX3 has been reported to induce TGF-β1 expression in the THP-1 macrophages [25]. In our study, treatment with rPTX3 also resulted in significantly elevated TGF-β1 levels in BALF in both asthma models. All the cytokines mentioned above have been proved to play essential role in asthma pathogenesis. Correspondingly, rPTX3 treatment also had a striking effect on AHR, as well as lung histopathology, as illustrated by the strong augmentation of peribronchiolar and perivascular inflammatory cell infiltration, mucus production, and collagen deposition, with significant increase in inflammatory scores, PAS scores, and collagen content, respectively. Accordingly, rPTX3 administration enhanced all aspects of the asthma phenotype seen in either OVA/Alum or OVA/LPS models.

Substantial evidences showed that IL-17A were significantly increased in the airways of patients with asthma, and closely correlated with main clinical parameters of asthma [26–30]. Meanwhile, IL-17A was shown to contribute to all asthma characteristics, including inflammatory cells infiltration [31], mucus hypersecretion, [32] tissue remodeling [33, 34], and AHR [35]. Notably, in the study by Balhara and colleagues, although increased Th17 regulators were shown in OVA-exposed PTX3 knockout mice compared with those in wild-type mice, including an increase in numbers of IL-6- and IL-23-producing dendritic cells, no significant difference in IL-17A concentrations in lung was observed [18]. Even more strikingly, in saline control group, PTX3 deficiency could confer a marked reduction in baseline IL-17A concentration of lung homogenates based on their reported results [18], which is in turn compatible with the increase in IL-17A in BALF by PTX3 administration in both asthma models observed in our study. Indeed, IL-17A was reported to exert an inhibitory effect on expansion of IL-17A-producing T cells, and extracellular release of the upstream regulator IL-23 through a negative feedback mechanism [36, 37]. Accordingly, as a potential explanation for the apparently contradictory result in the study by Balhara et al [18], it seems reasonable to presume that lower baseline IL-17A levels in PTX3 knockout mice contribute to enhanced upstream pathway of Th17 immune response through a negative feedback mechanism. More studies are needed further to investigate these possibilities.

Of note, although human genetic findings and most animal studies on the long pentraxin PTX3 suggest that PTX3 is important in conferring host resistance to infection, a prevalent concept has been promoted: depending on different disease context, PTX3 may elucidate distinct role roles in disease pathogenesis [38]. For instance, PTX3 was found to limit postischemic acute and chronic kidney injury [11], but exacerbate tissue inflammation after intestinal ischemia and reperfusion in mice [39]. Additionally, PTX3 was shown to accelerate lung injury in high tidal volume ventilation in mice [40], while PTX3 deficiency significantly increased the magnitude of lung injury induced by LPS [10]. In view of the complexity of PTX3 pathophysiology, it was recently considered as a key homeostatic component at the crossroad of innate immunity, inflammation, tissue repair, and cancer, reviewed by Garlanda et al [5]. It is tempting to speculate that PTX3, as a highly conserved pattern recognition molecule, may also act as a versatile homeostatic regulator in the context of allergic disease, and the disruption of this function by either PTX3 deficiency or treatment may further exacerbate the immune dysregulation in asthma.

With these data, we cannot evaluate the effect of rPTX3 on allergic sensitization, as the administration was only performed during challenge period. However, the effect of PTX3 administration was firstly evaluated in the context of allergic disease in our study, and the results showed that intranasal administration of rPTX3 significantly enhanced airway inflammation in both eosinophilic and neutrophilic asthma models. Thus, our study didn’t support PTX3 administration as a treatment option for asthma. Further study is still needed to provide a comprehensive understanding of the exact mechanism by which PTX3 regulates airway inflammation in asthma.

## Supplementary information

**Additional file 1 Figure S1.** Effects of rPTX3 treatment on serum total IgE. Data are shown as the mean ± SEM (*n* = 6–10). Data were analysed using one-way analysis of variance (ANOVA) followed by the Tukey-Kramer post hoc test for multiple comparisons.

## Data Availability

Not applicable.
