# Supplementary material for: Pentraxin 3 promotes airway inflammation in experimental asthma
Source: Respir Res. 2020 Sep 16;21:237. doi: 10.1186/s12931-020-01499-6 (PMC7493172; doi:10.1186/s12931-020-01499-6)
Supplement: Supplementary file 1 — Additional file 1 Figure S1. Effects of rPTX3 treatment on serum total IgE. Data are shown as the mean ± SEM (n = 6–10). Data were analysed using one-way analysis of variance (ANOVA) followed by the Tukey-Kramer post hoc test for multiple comparisons. [file 12931_2020_1499_MOESM1_ESM.zip › Supplementary material.docx]

**Supplementary Figure 1.** Effects of rPTX3 treatment on serum total IgE. Data are shown as the mean ± SEM (n = 6-10). Data were analysed using one‐way analysis of variance (ANOVA) followed by the Tukey‐Kramer post hoc test for multiple comparisons.
